# Investigation of the interactions and electromagnetic shielding properties of graphene oxide/platinum nanoparticle composites prepared under low-dose gamma irradiation

**DOI:** 10.1038/s41598-025-12655-7

**Published:** 2025-07-24

**Authors:** Dejan Kepić, Miloš Milović, Dušan Sredojević, Andjela Stefanović, Brankica Gajić, James L. Mead, Blaž Nardin, Blaž Likozar, Janvit Teržan, Muhammad Yasir, Warda Saeed, Svetlana Jovanović

**Affiliations:** 1 Vinča Institute of Nuclear Sciences - National Institute of the Republic of Serbia, University of Belgrade, Mihajla Petrovića Alasa 12-14, Belgrade, 11351 Serbia; 2 Faculty of Chemistry, University of Belgrade, Studentski trg 12-16, Belgrade, 11158 Serbia; 3 Department of Computing Science, University of Oldenburg, D-26129 Oldenburg, Germany; 4 Faculty of Polymer Technology, Ozare 19, Slovenj Gradec, 2380 Slovenia; 5 Department of Catalysis and Chemical Reaction Engineering, National Institute of Chemistry, Hajdrihova 19, Ljubljana, SI-1000 Slovenia

**Keywords:** Graphene oxide, Platinum nanoparticles, Composites, Gamma irradiation, Electromagnetic shielding, Density functional theory, Chemistry, Materials chemistry, Theoretical chemistry

## Abstract

A low-dose gamma irradiation was used for the one-step synthesis of graphene oxide/platinum nanoparticle composites. Various spectroscopic and microscopic methods were employed to structurally and morphologically characterize the prepared composites, and the nature of the interactions between graphene oxide sheets and platinum clusters was investigated using density function theory (DFT). Gamma irradiation caused the reduction of hexachloroplatinic acid, resulting in the formation of Pt nanoparticles and the simultaneous partial reduction of graphene oxide (GO). Pt nanoparticles synthesized at doses of 10 and 20 kGy showed a homogeneous GO surface coverage with a high portion of particles with sizes of up to 10 nm. The DFT results indicate a difference in electrical conductivity between GO and PtNPs. This could cause charge redistribution across the contact area, creating a conductive network at the interface that should enhance the EMI shielding capabilities of the composite. The shielding efficiency of the composites measured at the X band showed a blockage of 77% of the incident electromagnetic wave at a center frequency of 10 GHz. The composite prepared at a 20 kGy dose exhibited a greater contribution from a mismatch loss component, attributed to the improved electrical conductivity induced by irradiation.

**Supplementary Information:**

The online version contains supplementary material available at 10.1038/s41598-025-12655-7.

## Introduction

An increasing number of modern-day gadgets that make our lives easier saturate our environment with electromagnetic waves (EMW), causing electromagnetic wave pollution. The effects of EMW exposure on living organisms are still debatable, and it is speculated that low-frequency electromagnetic fields might affect sleep quality, increase stress levels, trigger depression and anxiety^1^, or even increase the risk of brain tumors^2^. On the other hand, the saturation of electromagnetic waves leads to electromagnetic interference (EMI), which can detract from the device’s performance and shorten its lifetime. To combat these issues, it is essential to develop materials that can protect people and instruments from the effects of electromagnetic waves. Typical EMI shielding materials include metals in the form of sheets or foams^3^, two-dimensional transition metal carbides, nitrides, and carbonitrides known as MXenes^4^, cement-based materials^5^, as well as various carbon-based materials, such as carbon nanotubes, graphene and its derivatives, and carbon fibers^6,7^.

Metals possess excellent electrical conductivity, which makes them very effective at reflecting and absorbing electromagnetic waves. Chen et al. developed copper-coated carbon fiber composites and reported an EMI shielding effectiveness of 83.1 dB, which was roughly 30 dB higher than that of pristine carbon fiber fabrics^8^. However, metals are heavy, which limits their applications in aerospace, automotive, portable electronics, and wearable devices. Another drawback is their susceptibility to corrosion and rigidity. Unlike metals, MXenes are lightweight and have better flexibility. This, combined with their excellent conductivity and a large surface area, provides high EMI shielding effectiveness even at low thickness. The ultrathin, free-standing films of Ti_3_C_2_T_x_ MXene/nanocrystalline cellulose composite exhibited an EMI shielding effectiveness of 44 dB^9^. In another work, Tan et al. prepared a 15 μm-thick polyimide/Ti_3_C_2_T_x_ composite film which exhibited an outstanding EMI shielding effectiveness of 37 dB in the X-band range^10^. But the widespread application of MXenes in EMI shielding materials is mainly limited by their cost.

An ideal EMI shielding material is elastic, thin, lightweight, durable, and stable. Graphene and its derivatives and composites fulfill these requirements and have recently become the subject of numerous studies in shielding materials^11^. Graphene oxide (GO) and reduced graphene oxide (rGO) offer some benefits over graphene in terms of cost-effectiveness and processability. GO, for example, is the oxidized form of graphene with a significant portion of oxygen-containing functional groups attached to the graphene sheets, making GO dispersible in water. Besides forming those functional groups, the oxidation of graphene also increases the bandgap and introduces defects in the sp^2^ domains of the graphene structure^12^. The structure might be partially recovered by reducing GO and obtaining rGO, which has fewer functional groups and higher electrical conductivity than GO. Similar to pristine graphene, both GO and rGO have large surface areas, which, in combination with the present functional groups, offer the possibility for covalent or noncovalent bonding of various molecules, polymer chains, or nanoparticles, thus creating hybrid nanocomposite materials that exhibit superior properties compared to their components^13^. Additionally, various graphene- and carbon-based materials were investigated for EMI shielding applications. Milenkovic et al. obtained composites of GO and silver nanowires (AgNWs) in different GO/AgNW ratios and reported EMI shielding effectiveness from 0.9 dB to 4.5 dB^14^. Silver nanoparticles anchored on electrochemically exfoliated graphene showed 32% of the power transmitted through the sample at 2 GHz^15^. In contrast, carbonized biowaste materials were able to block 78.5% of the incident electromagnetic wave^16^.

Recently, tremendous attention has been devoted to platinum nanoparticles due to their rich electronic structure, stability, outstanding thermal and electrical conductivity, and nontoxicity^17^. Due to their remarkable properties, platinum nanoparticles (PtNPs) are widely investigated for biomedical applications^18^, catalysis^19,20^, and sensor applications^21,22^, among others. Their properties are controlled by the fabrication process. Conventional chemical synthesis implies the reduction of PtNP precursors such as hexachloroplatinic acid (H_2_PtCl_6_) by different reducing agents that influence the size, shape, yields, and stability of the obtained PtNPs^17^. For example, by reducing hexachloroplatinic acid with sodium borohydride, it is possible to obtain PtNPs with sizes of around 6 nm^23^. Switching the reducing agent to formaldehyde or hydrogen leads to an increase in PtNP size^24^. Moreover, hydrazine^25^, formic acid^26^, or ascorbic acid^27^ can also be used for the reduction. Conventional chemical synthesis also implies the presence of a stabilizing agent that prevents overgrowth or agglomeration of PtNPs, such as ethylene glycol (EG) or poly(vinyl pyrrolidone) (PVP)^23,28^.

Radiolytic synthesis emerges as an alternative to conventional synthesis, offering benefits in terms of time and chemical consumption. Radiation enables the homogeneous generation of nuclei and the formation of pure metallic nanoparticles without residuals and by-products^29^. Wang et al. applied a gamma irradiation strategy to decorate multi-walled carbon nanotubes with uniform PtNPs for the electrocatalysts in fuel cell applications^30^. A similar pathway was followed to prepare PtNP/graphene aerogel for the catalytic reduction of 4-nitrophenol^31^, rGO-PtNP composite for fabrication of a counter electrode in dye-sensitized solar cells^32^, rGO-PtNP composite for supercapacitor electrodes^33^, the polygonal angle PtNPs anchored to N-doped rGO for the oxygen reduction reaction catalysis^34^, or rGO-PtNP nanocatalyst for the electro‑oxidation of methanol^35^. However, all the aforementioned procedures involve high doses of gamma irradiation ranging from 50 to 300 kGy.

Here, we employed low-dose gamma irradiation (1-20 kGy) for the synthesis of PtNPs anchored to GO sheets in a one-step procedure. With low irradiation doses, the study aims to prevent the creation of defects due to the knocking out and spattering of C atoms from the graphene structure^36^ and to preserve sp^2^ regions. Additionally, by applying low irradiation doses, nanoparticle synthesis becomes both time- and cost-effective, thereby contributing to its sustainability. The structural and morphological properties of the prepared GO-PtNP composites were thoroughly examined, and the nature of the interactions between graphene oxide sheets and platinum clusters was investigated using density function theory (DFT). Additionally, we examined the capability of composites to prevent the transmission of electromagnetic waves within the 8–12 GHz frequency range.

## Methods

### Synthesis of GO

A modified Hummer’s method was used to synthesize GO, as previously reported^37^. In short, concentrated sulfuric acid (23.3 mL, Carlo Erba Reagents) was mixed with graphite powder (1 g, KS6, TIMREX^®^) in an ice bath under stirring. Then, KMnO_4_ (3 g, Merck) was slowly added to the mixture, and the stirring was continued for 15 min. Next, 50 mL of water was added and the temperature of the reaction mixture was kept at 40 °C for 30 min and then at 90 °C for 15 min. After that time, the reaction mixture was poured into 170 mL of water which contained 5 mL of hydrogen peroxide (30 vol%, Carl Roth). GO flakes were precipitated by centrifugation and washed several times with MiliQ water until the pH of the supernatant was 7. The GO powder was dried in a vacuum oven at 80 °C.

### Synthesis of GO-PtNP composites

A stable GO water dispersion with a GO concentration of 1 mg mL^-1^ was prepared using an ultrasound bath. The dispersion (200 mL) was mixed with isopropyl alcohol (20 mL, Fisher Chemicals) and hexachloroplatinic acid hexahydrate (11.4 mg, Tokyo Chemical Industry Co.) under stirring. The mixture was then divided equally into three vials and argon was purged through each vial for 15 min, after which the vials were hermetically sealed. The vials were exposed to gamma-ray flux from the ^60^Co nuclide to receive 1, 10, and 20 kGy doses, respectively. After the irradiation, the samples were filtered to remove reaction byproducts through 0.45 μm pore size cellulose nitrate membranes (Whatman) and washed with MiliQ water. For the synthesis of PtNP in the absence of GO, hexachloroplatinic acid hexahydrate was dissolved in a water/isopropyl alcohol mixture to achieve a concentration of 1 × 10^-4^ M, and the mixture was split into two vials. After purging argon, the vials were irradiated at doses of 1 and 20 kGy, respectively.

### Characterization

The UV-Vis absorption spectra were recorded on the LLG-uniSPEC 2 spectrophotometer in the range of 200–800 nm. For the measurements, a small quantity of the dried material was dispersed in water, and spectra were recorded in quartz cuvettes at room temperature. The X-ray diffraction (XRD) measurements were performed on a Philips PW 1050 X-ray powder diffractometer using Ni-filtered Cu Kα radiation and Bragg-Brentano focusing geometry. The diffraction intensity was recorded in the 2θ range of 10-90° with a step size of 0.02° and a counting time of 5 s per step. Eva software was used for the determination of phase composition. The unit cell parameters of the platinum phase were extracted from XRD patterns by using Powder Cell software. The crystallite size (Xs) of precipitated Pt has been estimated from the half-width β_½_ of the (111) Pt peak, by using Scherrer’s Eq. 

1 $$Xs{\text{ }} = {\text{ }}0.9{\text{ }} \times {\text{ }}\lambda {\text{ }}/{\text{ }}\left( {\beta _{\raise.5ex\hbox{$\scriptstyle 1$}\kern-.1em/ \kern-.15em\lower.25ex\hbox{$\scriptstyle 2$} } \times {\text{ }}\cos \theta } \right)$$

and for the calculation of the interlayer distance d_00l_ between sheets of graphene oxide (GO) and reduced graphene oxide (rGO), Bragg’s law has been used,

2 $${d_{00l}}={\text{ }}\lambda {\text{ }}/{\text{ }}\left( {2{\text{ }} \times {\text{ }}\sin \theta } \right)$$

where λ is the wavelength of incident X-rays (1.542 Å) and θ is the Bragg angle. A transmission electron microscope (TEM) examination of the samples was carried out using a JEOL (Tokyo, Japan) JEM-2100 F with an acceleration voltage of 200 kV. The composite samples were dispersed in ethanol using an ultrasound bath and a drop of the mixture was placed on lacey carbon copper grids (200 mesh, Agar Scientific) and dried in the air. For PtNP samples prepared without GO, a few drops of water dispersion were placed on holey carbon copper grids (200 mesh, Agar Scientific) and dried in the air. The particle size distributions were calculated using DigitalMicrograph software (Gatan, Inc.). The microstructure and elemental mapping of the composites were investigated using a Field Emission Gun Scanning Electron Microscope (FESEM, Mira3 Tescan, Oxford, UK) at 20 kV electron energy in a high vacuum. EDS analysis was conducted using an INCAx-act LN2-free Analytical Silicon Drift Detector (Oxford Instruments, Oxford, UK), with the PentaFET^®^ Precision and AZtec software version 4.3. X-ray photoelectron spectroscopy (XPS) was used to analyse the chemical states of Pt and C. The measurements were performed with a PHI VersaProbe 3 AD (Phi, Chanhassen, US) equipped with a monochromatic Al Kα X-ray source. The resolution of the analyzer was 0.65 eV. A dual-beam neutralisation system (electrons and ions) was used to attenuate the charging of the sample. The resulting peak shift due to charge neutralisation was corrected by aligning the metallic Pt-4f peak to 71 eV. Survey spectra were recorded at a pass energy of 224 eV with a step size of 0.8 eV, while high-resolution spectra were recorded at a pass energy of 27 eV with a step size of 0.05 eV. Three sweeps were performed for the survey spectra and 30 sweeps for the high-resolution spectra. Spectral deconvolution was performed with the Multipak software. For the electromagnetic shielding efficiency measurements, dry samples were mixed with a sodium silicate resin to produce a homogeneous paste. The prepared GO-PtNP paste concentrations were 0.33 g mL^-1^. The pastes were deposited on a 0.2 mm-thick Plexiglass sheet covered with a paper mold and formed into 22.86 mm × 10.16 mm thin films so that the size corresponds to the inner dimensions of WR-90 waveguide adapters used for the measurements. The transmission coefficients in the X-band (8–12 GHz frequency range) were measured using a Rohde & Schwarz ZVA 24 Vector Network Analyzer (VNA, Munich, Germany).

### Computational details

All calculations were performed using the Gaussian 09 suite of programs. We used density functional theory (DFT) to examine the structural parameters, electronic structures, and interaction energies of Pt/GO composites. The Pt_55_, Pt_18_, and Pt_21_ clusters were employed to model the electronic structures of Pt nanoparticles and their {111} and {100} facets, respectively. To model graphene oxide (GO), we utilized the C_40_H_16_O_2_(OH)_2_ cluster, which possesses two epoxy and two hydroxyl basal groups. Using the B3LYP-D3 function^38,39^, in conjunction with the 6-31G(d, p) basis set for light atoms^40^ and the LANL2DZ basis set with pseudo potentials for Pt atoms, the ground-state geometries of the Pt_18_/C_40_H_16_O_2_(OH)_2_ and Pt_21_/C_40_H_16_O_2_(OH)_2_ adducts are optimized. The platinum atoms of the Pt_18_ and Pt_21_ clusters were frozen during the optimization process to preserve the crystal structures that represent (111) and (100) planes, while all atoms of the C_40_H_16_O_2_(OH)_2_ cluster were relaxed. The non-covalent interactions within the Pt_18_/C_40_H_16_O_2_(OH)_2_ and Pt_21_/C_40_H_16_O_2_(OH)_2_ systems are depicted through the interaction energies (E_int_), and counterpoise corrections are used to remove the basis set superposition error (BSSE). This computation technique makes use of the following equations:

3 $$\Delta {E_{\operatorname{int} }}={\text{ }}{E_{Pt18/21}}+{\text{ }}{E_{GO}}-{\text{ }}{E_{adduct}}$$

4 $$\Delta{E_{\operatorname{int} }}^{{CP}}={\text{ }}{E_{\operatorname{int} }}+{\text{ }}{E_{BSSE}}$$

The Mulliken charges and molecular electrostatic potentials (MEPs) are computed to illustrate the charge distributions on these composites. All these calculations are performed in the gas phase. *GaussView* software was used to obtain the molecular representation of the clusters, ESP maps, Mulliken color schemes, and FMO orbitals, whereas the *GaussSum* program was utilized for acquiring total and partial density of state (TDOS/PDOS) diagrams.

## Results and discussion

Platinum nanoparticles (PtNPs) were synthesized directly on graphene oxide (GO) sheets in a one-step process using low-dose gamma irradiation to the mixture of hexachloroplatinic acid and GO in water. During irradiation, the radiolysis of water generates free radicals, ions, and neutral molecules, with hydrated electrons (e⁻_(aq)_) and hydroxyl radicals (OH^•^) being the most abundant species^41^. The addition of isopropyl alcohol acts as a scavenger for oxidative species, such as hydroxyl radicals and hydrogen peroxide, which promotes the predominance of the reduction reaction. The reducing species formed during radiolysis, primarily hydrated electrons and hydrogen radicals (H^•^), reduce PtCl_6_^2-^ ions to metallic platinum (Pt^0^), resulting in the nucleation and growth of PtNPs^29^. Simultaneously, the irradiation partially reduces the GO, as indicated by a change in the color of the dispersions. While the sample irradiated at 1 kGy remained yellow-brown, the dispersions turned darker at doses of 10 and 20 kGy. It was reported that hydrated electrons show a high affinity towards carbonyl and carboxyl groups present in GO, which act as trapping centers for these electrons^42^. Hence, hydrated electrons can remove oxygen-containing groups from the GO surface, resulting in its reduction. Since the number of these electrons depends on the absorbed radiation dose, it is possible to control the degree of GO deoxygenation by selecting the appropriate dose^43^.

**Fig. 1:**
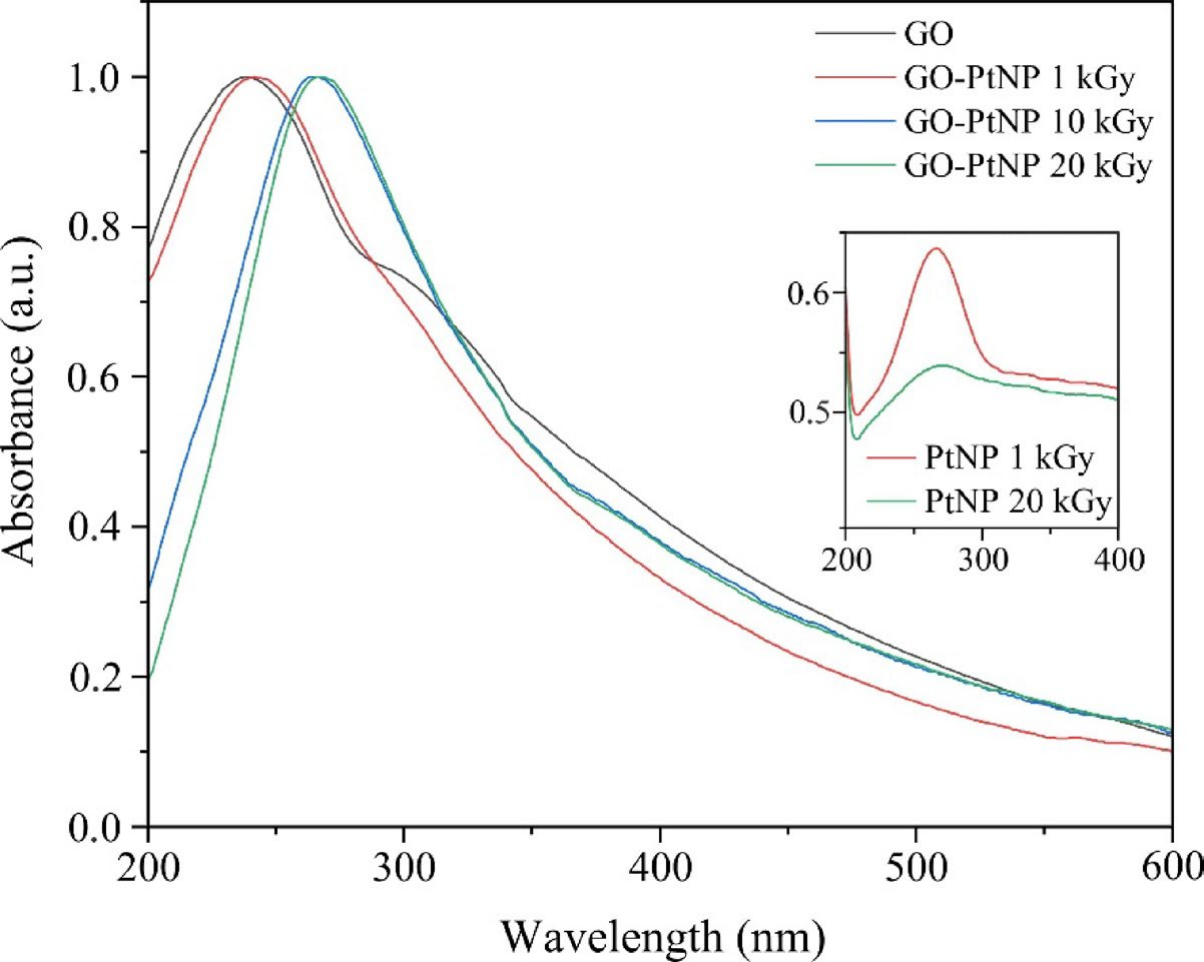
Normalized UV-Vis spectra of GO and GO-PtNP composites and PtNPs synthesized without GO (inset).

After irradiation, the UV-Vis spectra of the GO-PtNPs dispersed in water were recorded (Fig. 1). GO exhibits one broad, intensive peak at ~230 nm, resulting from the π-π* transition of aromatic C-C bonds, and one smaller feature at ~300 nm, originating from the n-π* transition of C-O bonds^44^. The reduction of GO under gamma irradiation, indicated by the change of color of the dispersions, initiates the gradual redshift of the 230 nm peak and the simultaneous disappearance of the 300 nm feature^45^. On the other hand, PtNPs have a surface plasmon resonance (SPR) peak that appears in the UV region^46,47^. As shown in Fig. 1, with an increase in the irradiation dose, the prevailing maximum shifts in position from 241 nm for the lowest to 268 nm for the highest applied dose. This shift occurs as a joint contribution of the redshift of the aromatic C-C bonds peak of GO and the occurrence of the SPR peak of PtNPs. To prove the contribution of PtNPs, we prepared PtNPs without GO at the doses of 1 and 20 kGy and recorded UV-Vis spectra (inset of Fig. 1). Both spectra show a broad peak centered at 266 nm, which is more prominent for the PtNPs prepared at 20 kGy.

**Fig. 2:**
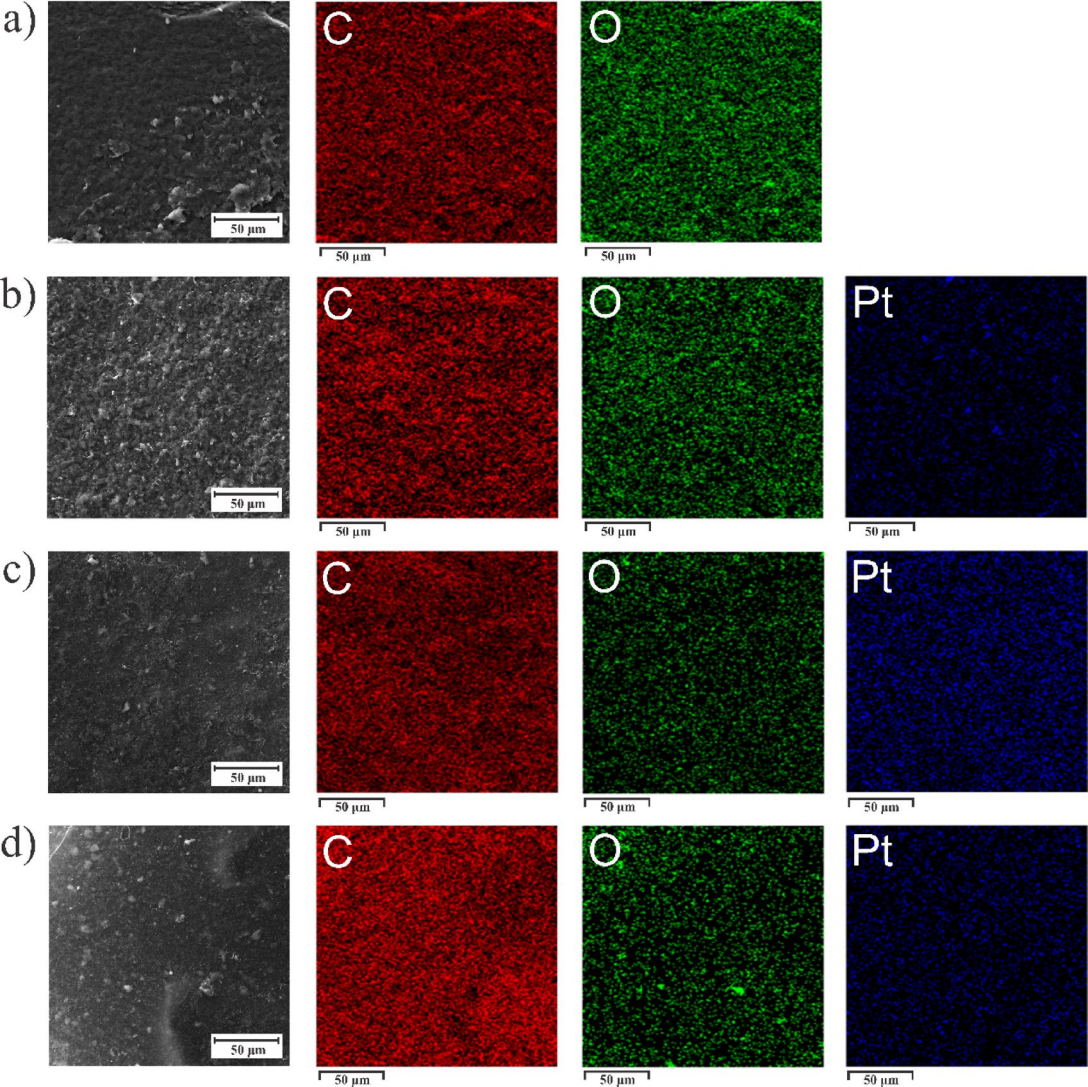
SEM image and the corresponding EDS elemental maps for C, O, and Pt for (**a**) GO, (**b**) GO-PtNPs prepared at 1 kGy, (**c**) GO-PtNPs prepared at 10 kGy, and (**d**) GO-PtNPs prepared at 20 kGy.

To inspect the morphology of the composites, we performed FESEM analysis (Fig. S1, Supporting Information). GO is present as stacked sheets with crumbled edges, which is an inherent characteristic of the dried GO sample. Irradiated samples show GO sheets covered with PtNPs. EDS elemental maps show the distribution of the constituent elements of the composites (Fig. 2). GO sheets are uniformly oxidized and, besides C and O, show trace amounts of S as a residual from the synthesis. GO-PtNP composites prepared at 10 and 20 kGy show homogeneous coverage of GO sheets with PtNPs. On the other hand, the GO-PtNP sample prepared at the lowest applied dose (1 kGy) exhibits islands with sparse populations of PtNPs and areas where PtNPs appear large and clustered. The weight and atomic percentages of the constituent elements of the GO and GO-PtNP composites are given in Table S1 (Supporting Information). The O/C ratio decreases with an increase in the irradiation dose, indicating a reduction in GO under gamma irradiation. The composite GO-PtNP prepared at 10 kGy showed the highest percentage of Pt.

**Fig. 3:**
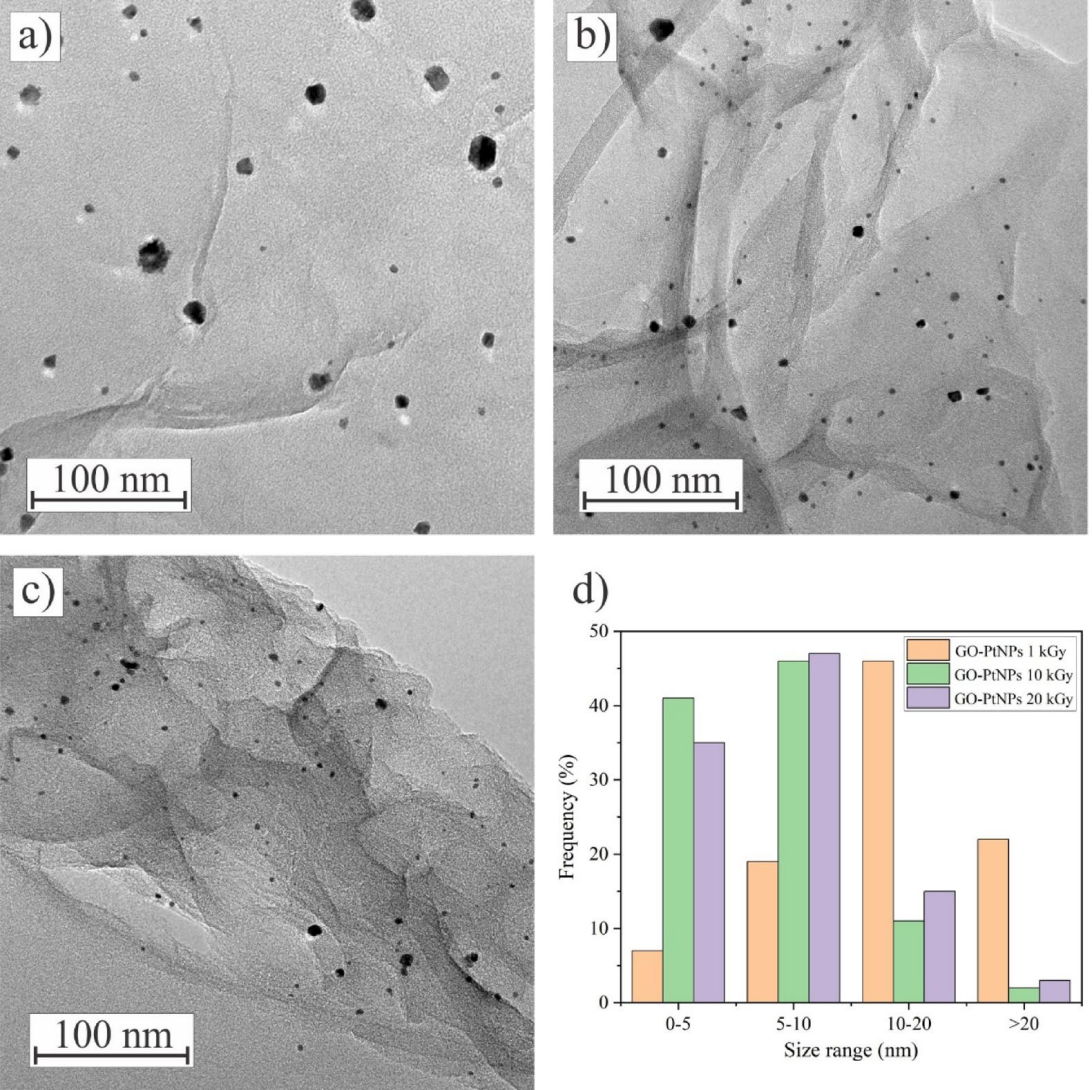
TEM images of (**a**) GO-PtNPs prepared at 1 kGy, (**b**) GO-PtNPs prepared at 10 kGy, (**c**) GO-PtNPs prepared at 20 kGy, and (**d**) particle size distribution of PtNPs synthesized at different irradiation doses.

Figure 3 represents the TEM images of GO-PtNP composites prepared at different irradiation doses. The images reveal the wrinkles and ripples of the GO sheets, with sporadic occurrences of folded edges. The difference in the atomic number between Pt and C enables a clear distinction of the formed PtNPs at the GO surface. PtNPs are mostly spherical, with a small portion of irregularly shaped particles. A detailed inspection of TEM images of the composite prepared at 1 kGy reveals the presence of agglomerates of PtNPs, which follows the EDS analysis. On the other hand, PtNPs synthesized at doses of 10 and 20 kGy appear to be homogeneously distributed over the GO sheets. The samples prepared at the lowest and the higher doses also differ in PtNP size distribution (Fig. 3d). While the majority of PtNPs synthesized at 1 kGy are bigger than 10 nm, nanoparticles synthesized at the higher doses are smaller and most of them have a size of less than 10 nm. The reduction in nanoparticles’ size with an increase in the irradiation dose has been reported previously^48–50^. The size dependence on the irradiation dose is guided by the two successive processes: nucleation and aggregation^29^. At lower irradiation doses, the number of nuclei generated is lower than the amount of available metal ions for growth, resulting in the formation of larger particles. Conversely, at higher doses, most of the precursor is used to produce a greater number of nuclei, exceeding the concentration of remaining unreduced ions, which in turn limits growth and yields smaller particles.

To illustrate the influence of GO on the formation of PtNPs, we followed the same experimental procedure in the absence of GO or other stabilizing agents, and the samples were irradiated at 1 and 20 kGy doses. For both the applied doses, PtNP agglomerates larger than 100 nm of undefined shape were noticed (Fig. S2, Supporting Information). Besides, the colloids of as-prepared PtNPs were unstable and tended to precipitate.

**Fig. 4:**
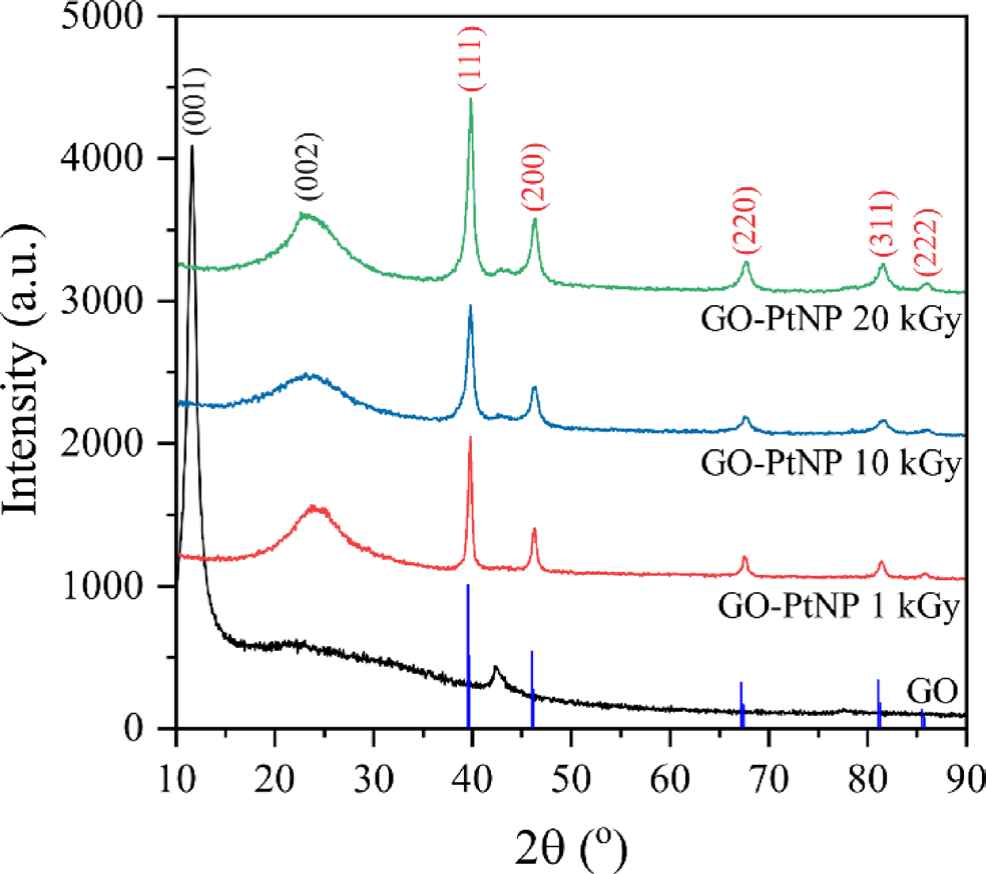
XRD spectra of GO and GO-PtNP composites with reflections of Pt reference card PDF # 04-802. GO peaks are indexed with black and Pt peaks with red indices.

Gamma irradiation creates a reductive environment that causes the precipitation of elemental Pt and the partial reduction of GO to rGO, as evidenced by XRD in Fig. 4. Pt crystallized within cubic *Fm-3m* s.g. no 225 (PDF no. 04-802) with the lattice parameter a ≈ 3.93 Å for all irradiated samples (Table S2, Supporting Information). The obtained Pt phase is nanocrystalline, as indicated by its broad XRD reflections, with an estimated crystallite size of around 20 nm for the PtNPs prepared at 1 kGy and around 14 nm for those prepared at 10 and 20 kGy. Moreover, by comparing estimated crystallite sizes with the particle sizes visible in Fig. 3 (TEM), one can deduce that observed Pt nanoparticles are monocrystals. In the XRD profile of the starting GO, the position of the (001) reflection at 2θ ≈ 11.6° corresponds to the interplanar distance of around 7.63 Å between basal planes of GO^51^. After gamma irradiation, the (001) peak disappears from the XRD patterns with the emergence of the new peak (002) at 2θ ≈ 23.4°, which corresponds to the interplanar distance of around 3.80 Å between the basal planes of partially reduced GO^52^. This interlayer shrinkage occurs due to the loss of graphene oxide’s functional groups after gamma irradiation, confirming its subsequent reduction^11^.

**Fig. 5:**
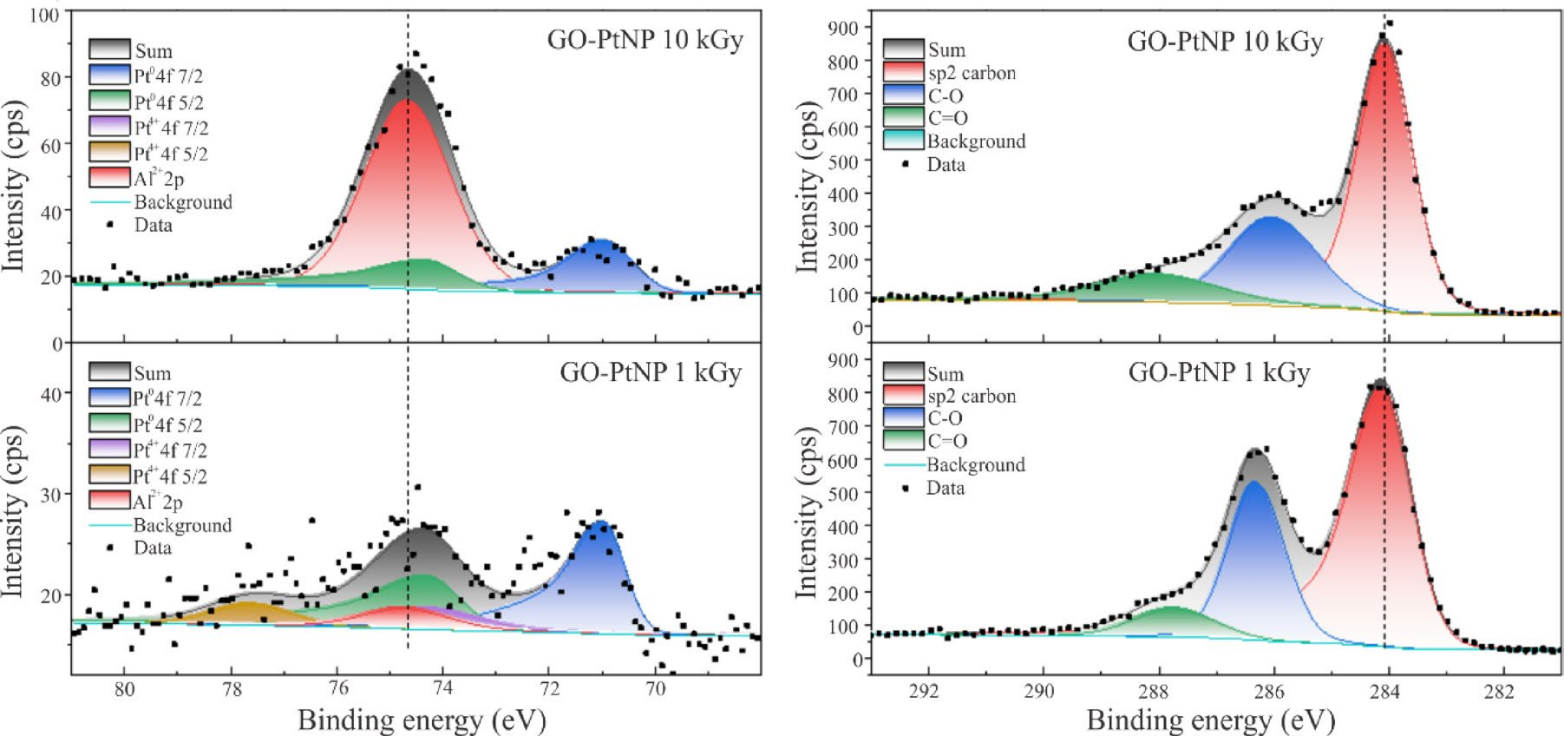
The Pt (left) and C (right) spectra from XPS analysis of GO-PtNP composites prepared at radiation doses of 1 kGy and 10 kGy. The alumina peak originates from the substrate.

To investigate the elemental composition, chemical state, and electronic environment, the composites were deposited on Al substrates and XPS analysis was performed (Fig. 5). As can be seen, the peaks for the Pt region align well with the literature data^53^. At a 1 kGy irradiation dose, approximately 20% of the platinum oxide is present, with the primary peak positioned at 74.3 eV. This is approximately 0.2 eV lower than the value reported in the literature^54^. This is expected due to the smaller particles being close to electronegative species, such as oxides^55^. The absence of XRD reflections of platinum oxide, as detected by XPS, indicates its amorphous nature. The carbon region shows a majority of sp^2^ hybridized carbon-carbon bonds, positioned at approximately 284 eV^56^. As expected, at the dose of 1 kGy, there is slightly more oxidized carbon present (~5%), with peaks positioned at approximately 286 and 288 eV, compared to the composite prepared at the higher dose. This correlates with the UV-Vis and EDS data.

### Computational analysis

The Pt_55_ cluster has been chosen as a benchmark for calculating electronic structures and interaction energies between GO and PtNPs. This cluster replicates a face-centered cubic lattice (*fcc*) of bulk platinum with a body-centered cuboctahedral pattern (O_h_). This makes it possible to use the complete O_h_ symmetry, which simplifies the computational procedure using a fully self-consistent DFT approach, with appropriate basis sets and computationally precise numerical integration.

The optimized structure of the Pt_55_ cluster is shown in Fig. S3a (Supporting Information), with two indicated crystalographic planes. The Pt_55_ cluster consists of five {100} layers stacked along the C_4_-axis, which include 9, 12, 13, 12, and 9 platinum atoms, with a Pt-Pt distance of 2.79 Å and an interplane distance of 1.97 Å. On the other hand, this cluster can also be viewed as stacked {111} surfaces, consisting of five consecutive {111} nano-flakes of 6, 12, 19, 12, and 6 platinum atoms with an interplane distance of 2.28 Å and overall radius of 1.1 nm. Fig. S3b combines different colors to show electrostatic potential maps (MEP) of the Pt_55_ cluster. Green indicates regions with zero potential, whereas red and blue display the molecules’ electron-rich (negative) and electron-deficient (positive) parts. The MEP reveals that the region around the cluster’s edges is electropositive (blue), while negative regions (red) are those placed at the facets. It should be stressed that {100} surfaces are more negatively charged than {111}. According to the calculated Mulliken charges, the platinum atoms at the exterior of the cluster are almost neutral, while those in the interior are positively charged (green atoms; Fig. S3b). On the other hand, the platinum atom at the cluster’s center is highly negatively charged (red atom). The calculated density of state (DOS) diagram of the Pt_55_ cluster shows its electronic structure, which proves the metallic character of PtNPs (Fig. S3c). A smooth conductivity of PtNPs may be suggested by the electronic states close to the Fermi level, with no gap between the valence and conduction bands.

To reduce the computational burden of calculating the interactions with GO, we used Pt_18_ and Pt_21_ clusters to model {111} and {100} surfaces of bulk platinum, respectively. The DOS diagrams of these clusters (Figs. S4a and S6a) are very similar to the larger Pt_55_ cluster, proving that the electronic structures are preserved. The Pt_18_ cluster comprises two stacked {111} flakes of 6 and 12 atoms, while Pt_21_ consists of two overlapped {100} planes with 9 and 12 Pt atoms. We used the C_40_H_16_O_2_(OH)_2_ cluster to mimic the GO surface rich in hydroxyl and epoxy groups. The corresponding density of states (DOS) diagram reveals a bandgap of 1.73 eV (Fig. S4b), close to the value of reduced GO. It could be further noticed that there are no gaps between the valence and conduction bands of the Pt_18_@C_40_H_16_O_2_(OH)_2_ and Pt_21_@C_40_H_16_O_2_(OH)_2_ adducts, according to the PDOS/TDOS diagrams in Figs. S4c, S6c. This may point to the increased conductivity of graphene oxide coated with platinum nanoparticles.

The computed Mulliken charges (Q_*Mulliken*_) of Pt_18_@C_40_H_16_O_2_(OH)_2_ and Pt_21_@C_40_H_16_O_2_(OH)_2_ complexes are presented in Figs. S5a, S7a, respectively. The results indicate charge transfers from GO to the Pt_18_ and Pt_21_ clusters. According to the Q_*Mulliken*_, Pt_18_ and Pt_21_ clusters accept electron densities of 0.44 and 0.31 *e*^-^, respectively. This is reasonable since the {111} surface is less negatively charged than the {100} surface and may allow for more negative charges. The electron-accepting nature of the P_18_ and Pt_21_ clusters within the Pt_18_/Pt_21_/GO systems is also suggested by the molecular electrostatic maps (MEPs) of the respective adducts (Figs. S5b and S4b). In both systems, the GO core is electropositive (blue), while the Pt clusters are negatively charged with the most negative region (red) on the contact surface area. It has been shown that the interfacial polarization, which originates from the difference in electrical conductivity of the materials in the interface, contributes to the loss of electromagnetic waves.

**Fig. 6:**
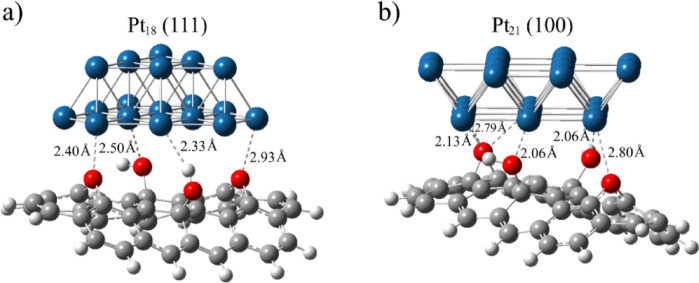
The optimized structures of (**a**) Pt_18_@C_40_H_16_O_2_(OH)_2_ and (**b**) Pt_21_@C_40_H_16_O_2_(OH)_2_ adducts with indicated noncovalent contacts.

**Table 1:** The calculated binding energy (Δ*E* and Δ*E*_CP_, kcal mol^-1^) between Pt_18_ (111) and Pt_21_ (100) clusters with C_40_H_16_O_2_(OH)_2_, with and without correction for BSSE.

	(111)−Pt_18_@C_40_H_16_O_2_(OH)_2_			(100)−Pt_21_@C_40_H_16_O_2_(OH)_2_		
	ΔE	BSSE	ΔE_CP_	ΔE	BSSE	ΔE_CP_
ωB97xD (D2)	−88.5	19.0	−69.5	−76.9	23.5	−53.4
B3LYP-D2	−110.8	23.5	−87.3	−102.9	28.1	−74.8
PBE0-D3	−69.7	20.2	−49.5	−68.7	25.2	−43.5
B3LYP-D3	−80.2	22.7	−57.5	−72.5	28.0	−44.5

To determine the strength of binding between GO and PtNPs, we used Pt_18_/Pt_21_@C_40_H_16_O_2_(OH)_2_ adducts and re-optimized them by freezing the structures of Pt clusters. The optimized geometries of these systems are presented in Fig. 6. Strong interactions are indicated by the short contacts (<2.5 Å) between Pt atoms and oxygen atoms from epoxy and hydroxyl groups. The interaction energies that quantify interfacial bonding between Pt_18/21_ and GO clusters are computed with several functionals, including Grimme’s dispersion corrections (GD2 & GD3). The basis set superposition error (BSSE) was removed by using counterpoise correction. All these values are summarized in Table 1. The results obtained with different functionals suggest that the GO cluster interacts stronger with {111} than with {100} surface. This is because the {100} plane is more negatively charged than {111}, causing somewhat stronger electrostatic repulsions with negatively charged oxygen atoms at the GO surface. In addition, the interaction energies obtained with dispersion-corrected functionals (GD2) are overestimated compared to the more accurate GD3 corrections for accounting dispersion energies (Table 1). Nevertheless, the binding energies of -57.5 and -44.5 kcal mol^-1^, calculated for {111} and {100} planes at the B3LYP-D3 level, suggest significant noncovalent binding between GO and platinum nanoparticles.

The primary cause of interfacial polarization is the electrical conductivity difference between GO and PtNPs at the interface, which allows charge redistribution across the contact area. This is evident from the density of states (DOS) near the Fermi level (Figures S4 and S6) of Pt_18/21_ and GO, indicating the differing conductivity of the two materials. As a result, a conductive network is created at the interface, improving the EMI shielding capabilities of this composite.

### EMI shielding measurement

The scattering parameters of the GO-PtNP composite films deposited on a Plexiglas sheet were measured in the X-band frequency. The EMI shielding performance of the composites was evaluated using the waveguide method by placing the sample between the two WR-90 waveguide adapters and then measuring the S-parameters. According to the work by McDowell^57^, the mismatch loss is defined at a reference plane between a source and a load as the ratio of the power that would be delivered to a conjugate-matched load to the power delivered to the actual load. This parameter was calculated from the S_11_ parameter using the following equation:

5 $${L_M}_{{}}={\text{ }} - 10{\log _{10}}\left( {1 - {{\left| {{S_{11}}} \right|}^2}} \right)$$

Similarly, the efficiency of a transmission line segment is defined as the ratio of the power delivered to the load to the net input power. Accordingly, the dissipation loss of the shield is defined as the reciprocal of this efficiency. Considering the reflection coefficient (S_11_) and the transmission coefficient (S_21_), the dissipation loss can be expressed as:

6 $${L_D}={\text{ }} - 10{\log _{10}}\left( {{{\left| {{S_{21}}} \right|}^2}/\left( {1 - {{\left| {{S_{11}}} \right|}^2}} \right)} \right)$$

The shielding effectiveness (SE) of the material was expressed as the sum of the dissipation loss and mismatch loss:

7 $$SE{\text{ }}={\text{ }}{L_D}+{\text{ }}{L_M}$$

**Fig. 7:**
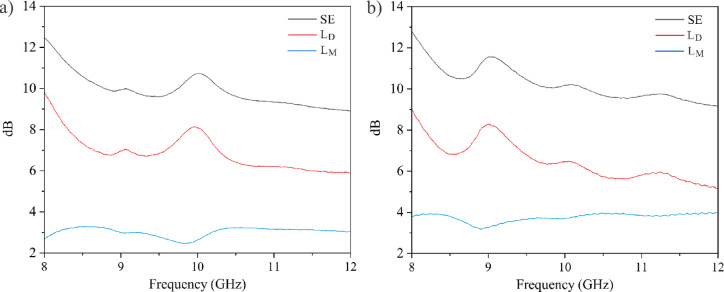
L_D_, L_M_, and SE values for (**a**) GO-PtNPs prepared at 1 kGy and (**b**) GO-PtNPs prepared at 20 kGy.

The calculated dissipation loss, mismatch loss, and shielding effectiveness of GO-PtNP composites prepared at doses of 1 and 20 kGy are plotted in Fig. 7.

Previous reports have shown that pristine graphene-based materials show relatively poor shielding performance in the corresponding frequency range^15^. The reduced GO showed a negligible shielding effect throughout the 8–12 GHz range. In contrast, the reduced electrochemically exfoliated graphene allowed transmission of 91.4% of the incident wave and simultaneously had only 8.6% of the reflection. Compared to pristine GO, GO-PtNP composites showed improved SE. An average EMI SE in the whole range for the sample prepared at 1 kGy was 9.89±0.77 dB (67.79±2.72%), while for the one at 20 kGy it was 10.28±0.81 dB (69.13± 2.76%). At a center frequency of 10 GHz, both samples reached the SE values of 11 dB, which corresponds to a blockage of 77% of the incident electromagnetic waves. From Fig. 7, it is evident that the shielding offered by the dissipative components is higher than that due to the mismatch losses of the sample. However, the total shielding effectiveness remains almost the same for both samples. This implies that both samples are dissipative in nature. Although both composites had similar SE values, the GO-PtNP sample prepared at 20 kGy showed an increased contribution of a mismatch loss component, implying a better electrical conductivity of this composite. The improved conductivity arose due to radiation-induced partial restoration of graphene’s sp^2^ structure.

## Conclusion

A one-step synthesis method for GO-PtNP composites was achieved using low-dose gamma irradiation. This process reduced hexachloroplatinic acid, leading to the formation of Pt nanoparticles, while simultaneously causing a partial reduction of GO. PtNPs produced at irradiation doses of 10 and 20 kGy displayed uniform coverage across the GO surface, with a significant proportion of particles measuring up to 10 nm in size. Additionally, gamma irradiation partially restored the sp² carbon structure of graphene. DFT analysis revealed differences in electrical conductivity between GO and PtNPs, suggesting that charge redistribution at their interface could establish a conductive network, thereby boosting the composite’s EMI shielding performance. The GO-PtNP composites prepared under low-dose gamma irradiation demonstrated a notable electromagnetic shielding effectiveness in the X band, blocking up to 77% of incident electromagnetic waves at a center frequency of 10 GHz. These findings suggest that such composites can serve as efficient and reliable protective materials for sensitive microelectronics, shielding them from unwanted electromagnetic interference. The results reported here are expected to broaden the availability of lightweight and cost-effective shielding materials.

## Supplementary Information

Below is the link to the electronic supplementary material.

Supplementary Material 1

## Data Availability

Datasets analyzed in the current study are available in the Zenodo repository (https://doi.org/10.5281/zenodo.14186519).
