# Supplementary material for: Investigation of the interactions and electromagnetic shielding properties of graphene oxide/platinum nanoparticle composites prepared under low-dose gamma irradiation
Source: Sci Rep. 2025 Jul 24;15:26924. doi: 10.1038/s41598-025-12655-7 (PMC12289968; doi:10.1038/s41598-025-12655-7)
Supplement: Supplementary file 1 — Supplementary Material 1 [file 41598_2025_12655_MOESM1_ESM.docx]

– Supporting Information –

Investigation of the Interactions and Electromagnetic Shielding Properties of Graphene Oxide/Platinum Nanoparticle Composites Prepared under Low-Dose Gamma Irradiation

Dejan Kepić^1,*^, Miloš Milović^1^, Dušan Sredojević^1^, Andjela Stefanović^1,2^, Brankica Gajić^1^, James L. Mead^3^, Blaž Nardin^4^, Blaž Likozar^5^, Janvit Teržan^5^, Muhammad Yasir^3^, Warda Saeed^3^, Svetlana Jovanović^1^

^1^Vinča Institute of Nuclear Sciences - National Institute of the Republic of Serbia, University of Belgrade, Mihajla Petrovića Alasa 12-14, 11351 Belgrade, Serbia

^2^Faculty of Chemistry, University of Belgrade, Studentski trg 12-16, 11158, Belgrade, Serbia

^3^Department of Computing Science, University of Oldenburg, D-26129 Oldenburg, Germany

^4^Faculty of Polymer Technology, Ozare 19, 2380 Slovenj Gradec, Slovenia

^5^Department of Catalysis and Chemical Reaction Engineering, National Institute of Chemistry,

Hajdrihova 19, SI-1000 Ljubljana, Slovenia

^*^Corresponding author. Tel.: +381 (0)11 34 08 582. E-mail address: d.kepic@vin.bg.ac.rs (D. Kepić).


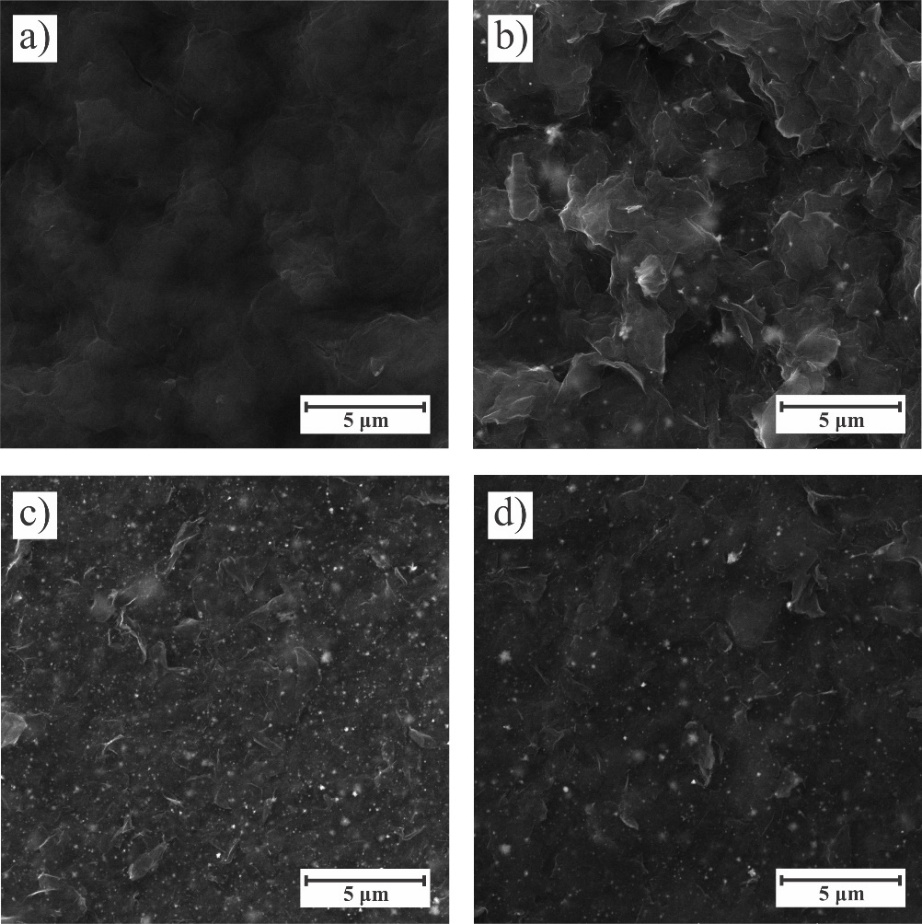


**Fig. S1.** FESEM images of (**a**) GO, (**b**) GO-PtNPs prepared at 1 kGy, (**c**) GO-PtNPs prepared at 10 kGy, and (**d**) GO-PtNPs prepared at 20 kGy.


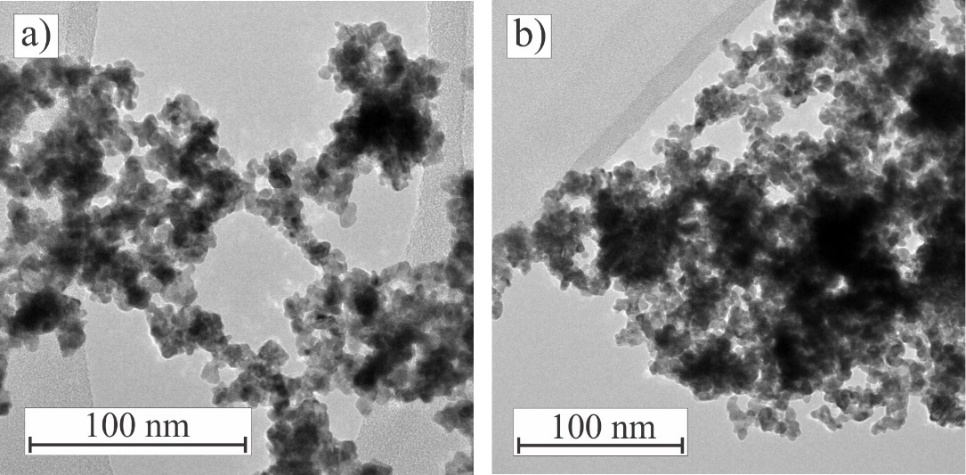


**Fig. S2.** TEM images of PtNPs prepared without GO at (**a**) 1 kGy and (**b**) 20 kGy.


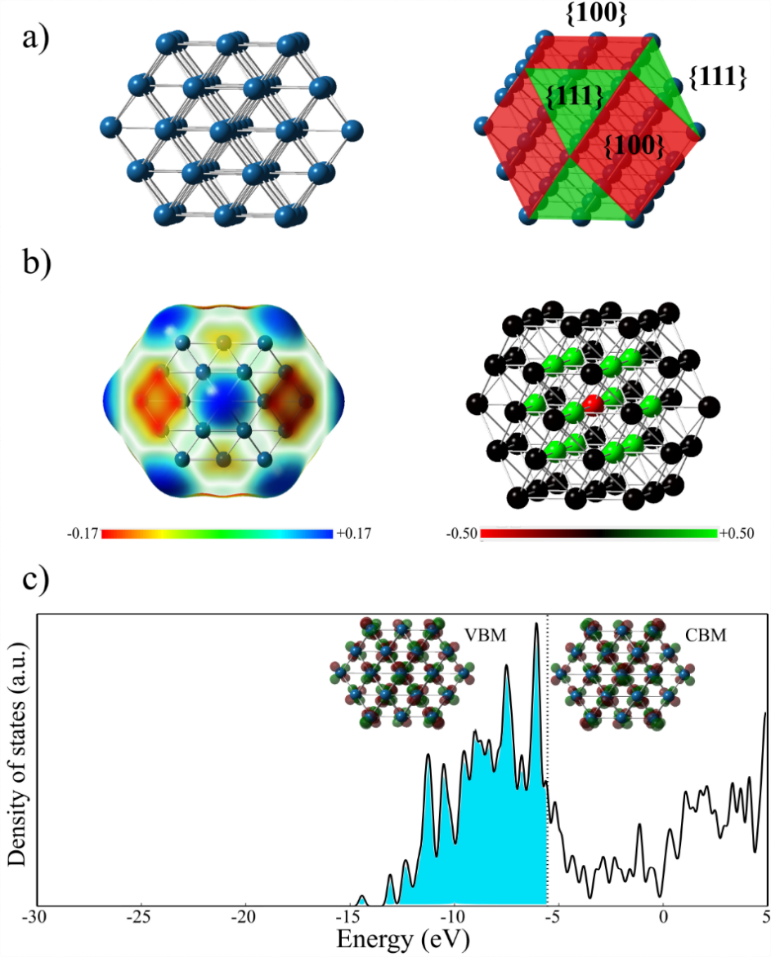


**Fig. S3.** (**a**) Pt_55_ cluster with the two indexed crystallographic planes, (**b**) ESP map and Mulliken charges, and (**c**) DOS diagram with the frontier molecular orbitals.


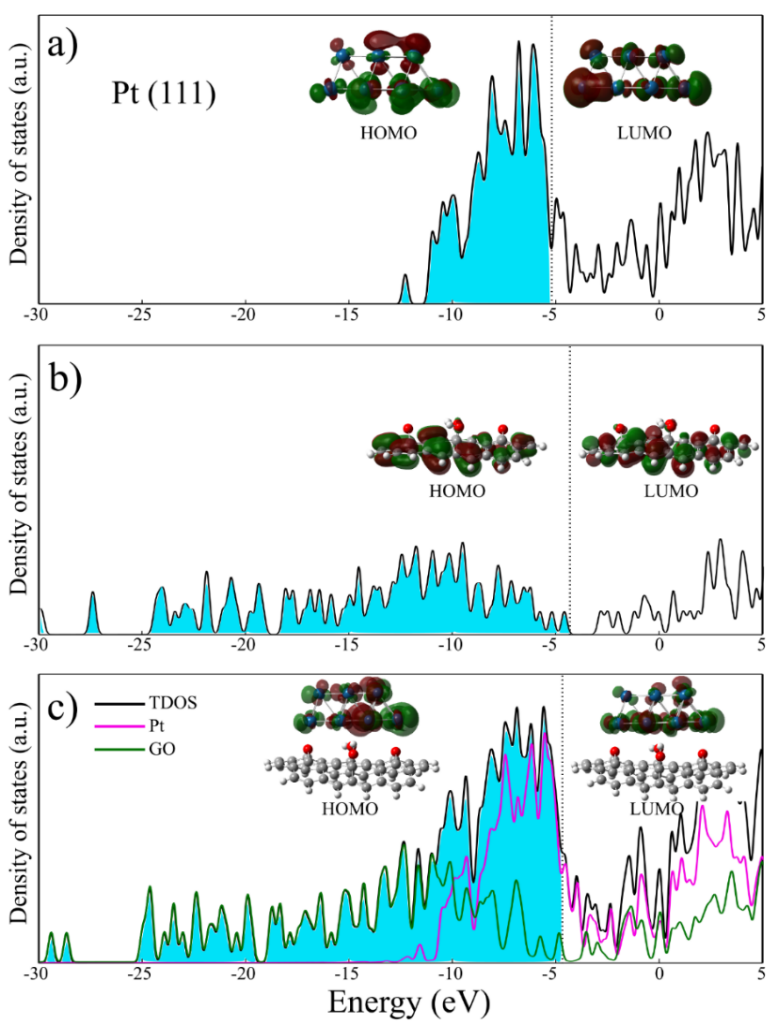


**Fig. S4.** The total and partial density of states (TDOS/PDOS) diagrams of (**a**) Pt_18_ (111), (**b**) C_40_H_16_O_2_(OH)_2_, and (**c**) Pt_18_@C_40_H_16_O_2_(OH)_2_ clusters with the frontier molecular orbitals.


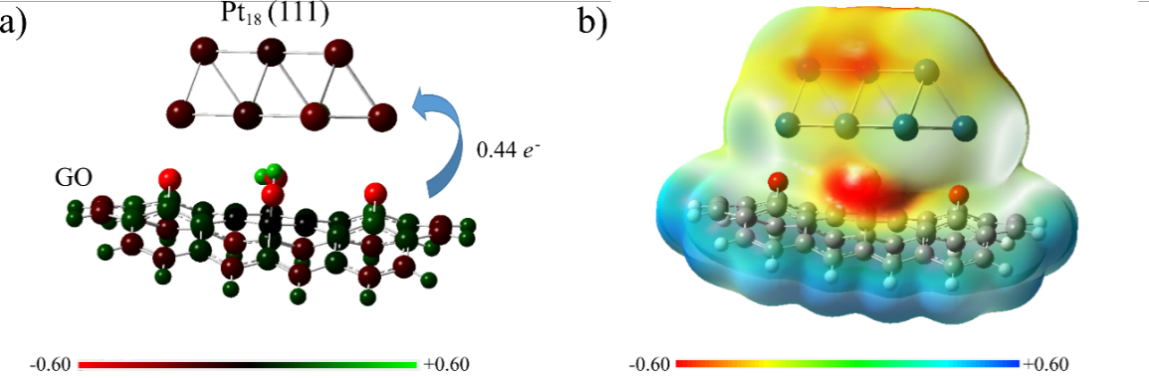


**Fig. S5.** (**a**) Mulliken charges and (**b**) the electrostatic potential map (MEP) of Pt_18_@C_40_H_16_O_2_(OH)_2_ adduct.

**
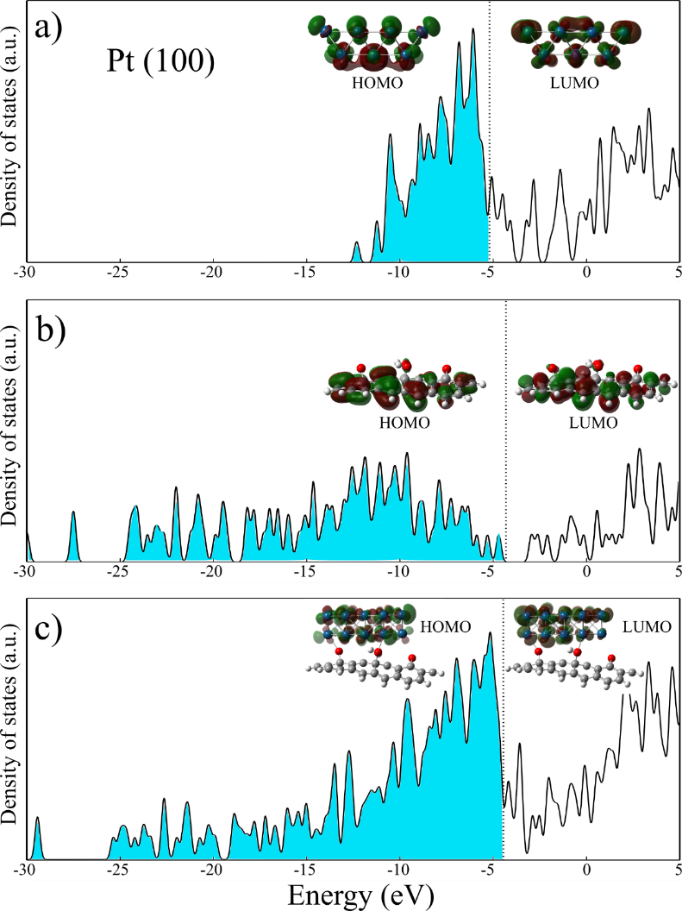
**

**Fig. S6.** The total density of state (DOS) diagrams of (**a**) Pt_21_ (100), (**b**) C_40_H_16_O_2_(OH)_2_, and (**c**) Pt_21_@C_40_H_16_O_2_(OH)_2_ clusters with the assigned frontier molecular orbitals.


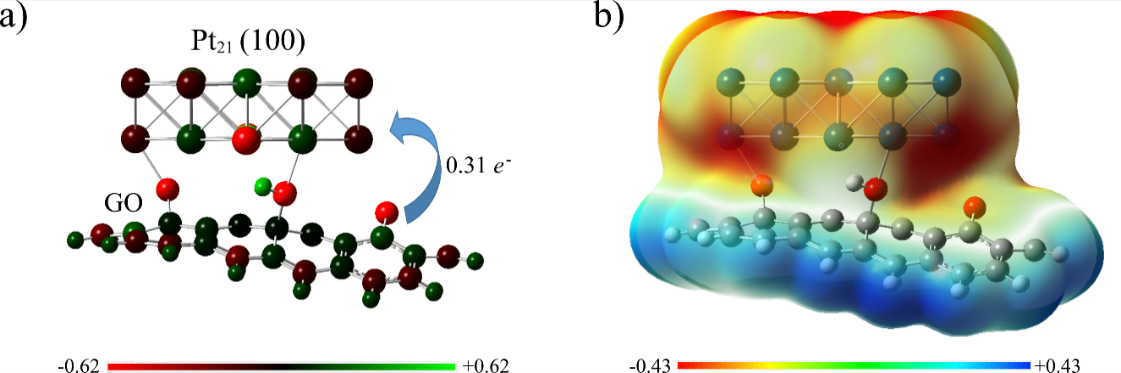


**Fig. S7.** (**a**) Mulliken charges and (**b**) the electrostatic potential map (MEP) of Pt_21_@C_40_H_16_O_2_(OH)_2_ adduct.

**Table S1.** Weight and atomic percentage of the constituent elements of the GO and GO-PtNP composites.

| **Sample:** | **Wt.%** | | | **At.%** | | |
| --- | --- | --- | --- | --- | --- | --- |
|  | **C** | **O** | **Pt** | **C** | **O** | **Pt** |
| GO | 54.3 ± 0.5 | 43.6 ± 0.5 |  | 61.9 | 37.4 |  |
| GO-PtNP 1 kGy | 59.8 ± 0.4 | 32.7 ± 0.4 | 3.8 ± 0.2 | 69.7 | 28.6 | 0.3 |
| GO-PtNP 10 kGy | 72.7 ± 0.4 | 19.0 ± 0.4 | 6.4 ± 0.2 | 82.6 | 16.2 | 0.4 |
| GO-PtNP 20 kGy | 76.7 ± 0.4 | 18.3 ± 0.4 | 2.9 ± 0.2 | 84.0 | 15.1 | 0.2 |

**Table S2.** The unit cell parameter and crystallite size of precipitated Pt (*Fm*-3*m*).

| **Sample** | **a (Å)** | **Cell volume (Å^3^)** | **Xs_111_ (nm)** |
| --- | --- | --- | --- |
| GO-PtNP 1 kGy | 3.926 | 60.5 | 20 |
| GO-PtNP 10 kGy | 3.927 | 60.6 | 14 |
| GO-PtNP 20 kGy | 3.925 | 60.5 | 14 |
